# Changes in inequality of childhood morbidity in Bangladesh 1993-2014: A decomposition analysis

**DOI:** 10.1371/journal.pone.0218515

**Published:** 2019-06-19

**Authors:** Rashidul Alam Mahumud, Khorshed Alam, Andre M. N. Renzaho, Abdur Razzaque Sarker, Marufa Sultana, Nurnabi Sheikh, Lal B. Rawal, Jeff Gow

**Affiliations:** 1 Health Economics and Policy Research, School of Commerce, Faculty of Business, Education, Law and Arts, Centre for Health, Informatics and Economic Research, University of Southern Queensland, Toowoomba, Queensland, Australia; 2 Health Economics and Financing Research, International Centre for Diarrhoeal Disease Research, Dhaka, Bangladesh; 3 School of Social Science and Psychology, Western Sydney University, Sydney Australia; 4 Department of Management Science, University of Strathclyde Business School, Glasgow, United Kingdom; 5 School of Health & Social Development, Deakin University, Melbourne, Australia; 6 School of Accounting, Economics and Finance, University of KwaZulu-Natal, Durban, South Africa; Institute of Economic Growth, INDIA

## Abstract

**Introduction:**

Child health remains an important public health concern at the global level, with preventable diseases such as diarrheal disease, acute respiratory infection (ARI) and fever posing a large public health burden in low- and middle-income countries including Bangladesh. Improvements in socio-economic conditions have tended to benefit advantaged groups in societies, which has resulted in widespread inequalities in health outcomes. This study examined how socioeconomic inequality is associated with childhood morbidity in Bangladesh, and identified the factors affecting three illnesses: diarrhea, ARI and fever.

**Materials and methods:**

A total of 43,860 sample observations from the Bangladesh Demographic and Health Survey, spanning a 22-year period (1993–2014), were analysed. Concentration curve and concentration index methods were used to evaluate changes in the degree of household wealth-related inequalities and related trends in childhood morbidity. Regression-based decomposition analyses were used to attribute the inequality disparities to individual determinants for the three selected causes of childhood morbidity.

**Results:**

The overall magnitude of inequality in relation to childhood morbidity has been declining slowly over the 22-year period. The magnitude of socio-economic inequality as a cause of childhood morbidity varied during the period. Decomposition analyses attributed the inequalities to poor maternal education attainment, inadequate pre-delivery care, adverse chronic undernutrition status and low immunisation coverage.

**Conclusions:**

High rates of childhood morbidity were observed, although these have declined over time. Socio-economic inequality is strongly associated with childhood morbidity. Socio-economically disadvantaged communities need to be assisted and interventions should emphasise improvements of, and easier access to, health care services. These will be key to improving the health status of children in Bangladesh and should reduce economic inequality through improved health over time.

## Introduction

During the 2000–2015 Millennium Development Goals (MDGs) reporting period, the global under-five mortality rate declined by more than half (from 90 to 43 deaths per 1,000 live births between 1990 and 2015) and the rate of reduction more than tripled globally when data from the early 1990s are taken into account [1]. Despite the impressive improvements, there were regional variations and in many of these, the trends were not sufficient to meet the MDG target [2,3]. Southern Asia was one of the regions found to have both a high rate of under-five mortality (at 50 deaths per 1,000 live births at the end of the MDG period) and a large number of total deaths [4,5]. The MDG report estimated that the global advance in child survival eludes many of the most vulnerable children, and the majority of them will perish from preventable causes, such as pneumonia, diarrhea, acute respiratory infection, and malaria [1]. Therefore, it is no surprise that diarrheal infection, acute respiratory infection (ARI), and fever account for approximately 33% of under-five mortality globally [6,7].

In Bangladesh, while the number of diarrhoea-related childhood deaths has dropped substantially, under-five morbidity rate has remained persistently high [8,9]. Globally, under-five deaths due to ARI has reduced from 1.7 million in 2000 to 0.94 million in 2013 [10]. However, the burden of ARI remains persistently high in Bangladesh (21% ARI-related mortality) [11], with approximately 50,000 children dying annually from ARI with a high pediatric hospitalisation rate of 40% [12–14]. Furthermore, ARI alone was accountable for 39.8 per 1000 children in 2013 [15]. Similarly, malaria (10.3%) [16], dengue fever (12.5%) [17], chikungunya (19%) [18], and typhoid are also common causes of childhood illness [12–15,19,20]. Bangladesh has already made improvements in the child health-related parameters of its people [21]; however, the prevalence of childhood morbidity is still distressingly high and widespread [22]. Poor health is often associated with low socioeconomic status and an inability to access health services, especially in rural communities [23]. One of the major challenges for potential improvement is to address the high levels of inequality in the distribution of healthcare across regions and socioeconomic groups [24,25]. The United Nations General Assembly’s recently adopted Sustainable Development Goals (SDGs) are aimed at fighting against inequality so that everybody gets similar access to, and benefits of, any health policy [26]. However, to achieve the health-related goals of SDG-3 (good health and well-bring) and SDG-10 (reduced inequalities especially access to health services) [26], it is essential to measure and address inequality so that all strata of society can potentially benefit.

The high disease burden of childhood morbidity in Low- and Middle-Income Countries (LMICs), including in Bangladesh, means there is a need for a better understanding of how childhood morbidity is distributed across socio-economic strata, which is crucial for adopting particular public health policies and designing effective health interventions [27–29]. The present study contributes to a recent body of work that examines the trends and degrees of inequality in terms of childhood morbidity, and decomposes the risk factors that better explain observed dynamics and contribute to change. Previous studies have indicated that a reduction in socio-economic inequalities has occurred at the same time as a decline in the prevalence of childhood morbidity at the national level [6,8,30]. However, uncertainty remains as to what factors are influencing both socio-economic inequalities and childhood morbidity trends. Therefore, the aim of this study was to investigate childhood morbidity trends over the last 22 years in Bangladesh and decompose changes to identify risk factors that explain the observed dynamics.

Examination of the key drivers of inequalities in childhood morbidity may offer insights into identifying priority health interventions both within and beyond the current health system. These inequalities are impeding the achievement of universal childhood health coverage. Additionally, significant findings are discussed in the context of national health policy in Bangladesh, and the experience of large-scale health interventions that might directly or indirectly contribute to reductions in childhood morbidity and mortality, especially amongst socio-economically disadvantaged children and their families.

## Materials and methods

### Data sources and sample

**Fig 1:**
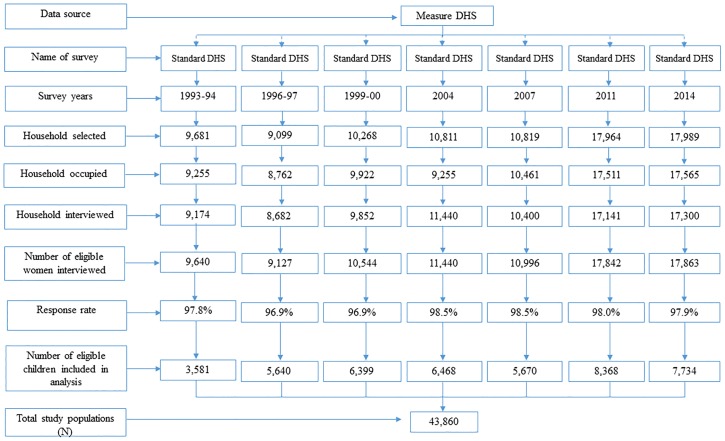
Distribution of study sample (N = 43,860).

The degree of socio-economic inequality in relation to childhood morbidity was examined using the latest seven rounds of the Bangladesh Demographic and Health Surveys (BDHS) from the 1993–94, 1996–97, 1999–00, 2004, 2007, 2011 and 2014 survey rounds [31–36]. Data were collected by Measure DHS retrospectively using structured quantitative survey tools from mothers whose youngest children were under-five years of age, with an average 98% response rate (Fig 1). The sampling technique, survey design, instruments, measuring system, data validity, reliability, and quality control are described elsewhere [31–36]. As per standard DHS practices, written consent was collected from all potential respondents before conducting the survey. The DHS dataset is publicly available; however, mailed consent also occurred as part of the protocol. Inconsistent, unusual/abnormal and missing observations were excluded from the analysis. Finally, a total of 43,860 sample observations were included in the analyses. The survey year and sample data are shown in (Fig 1).

### Definition of variables

#### Outcome variable

The outcome variables were the numbers of diarrheal episodes, symptoms of ARI (whether any cough or chest-related breathing difficulties were observed as a proxy for pneumonia), and fever for the youngest children under five years old, which occurred in the two weeks preceding the survey [31–36]. These constituted binary variables denoting one if present and zero if absent.

#### Independent variables

This study selected explanatory variables based on prior studies, epidemiological information, review of published demographic studies and the information in the BDHS datasets [6,12,14,29,30,31–38]. Individual-level factors, such as child’s age, squared age of child, sex of child, maternal age and educational status, birth order, preceding birth intervals, frequency of antenatal care visits, and households size were considered as potential factors in the analyses.

Chronic childhood undernutrition status was defined as “yes” if a child had experienced any adverse outcomes in terms of underweight, stunted, and wasting, or “no” otherwise. These adverse child health outcomes was measured based on the World Health Organization’s Child Growth Standard Guideline [39]. Mass media communication was defined as “yes” if the household had assess to electronic media, or broadcasting or newspaper, or “no” otherwise. Breastfeeding practice was grouped as “yes” if the child’s mother had experience of feeding breast milk, or “no” if no. Access to health facility was defined as “yes” if the household had an access to health services or health-related information, or “no” otherwise.

Child vaccination coverage was defined as “yes” if the child had vaccinated or “no” if non-vaccinated. Location of residence was considered rural or urban. Socioeconomic factors were presented by household wealth index, a composite score constructed from the asset vector of the household, as provided by BDHS and based on the ownership of 26 selected items of durable assets, including televisions and bicycles, materials used for housing construction, and types of drinking water sources, and sanitation facilities. Each household’s characteristics (assets) were dichotomised (‘yes’ if present and ‘no’ if not). Principal component analysis (PCA) was performed using the ownership of durable assets of households [40]. Weights were estimated by factor scores derived from the first principal component in the PCA. The constructed wealth score values were then assigned to individuals based on variables included in the model. The wealth score was divided into five groups based on overall asset ownership: poorest (Q_1:_ lowest 20%), poorer (Q_2_), middle (Q_3_), richer (Q_4_), and richest (Q_5:_ top 20%). These variables were used to examine the magnitude of inequalities in childhood diarrhea, ARI, and fever as well as changes in equality measures, comparing 1993/94 (1996/97 for fever because of a paucity of data) and 2014.

### Estimation strategies

#### Measuring and decomposing socio-economic inequalities

For the inequality analysis, comparisons of childhood morbidity were performed across wealth quintiles over the period specified. The absolute and relative differences (e.g., poor-rich difference and poor-rich ratio) in the prevalence of diarrhoea, ARI, and fever were calculated between the richest and poorest quintiles to capture the magnitude of the inequality between the two groups. However, these measures do not demonstrate the overall distribution of childhood morbidity in relation to key household socio-economic characteristics without adjusting for significant confounders. The standard measures of concentration index (CI) were used to examine the magnitude of household wealth-related inequality and the trends in childhood morbidity changes during the period of 1993/94 to 2014. The CI was derived based on the concentration curve (CC). The CC was constructed using the cumulative proportion of under-five childhood morbidity against the cumulative proportion of the population ranked from the poorest to the richest [41]. The estimated CI summarizes information contained in each CC [42]. The CI was derived from these curves as twice the area between the concentration curve and the line of equality (i.e., the 45° line, which characterizes a perfectly equal distribution of childhood morbidity among participants across the wealth index). The CC lies above (below) the line of equality if the under-five childhood morbidity is higher (lower) in values amongst the poorest. The CI can be estimated as the covariance between childhood morbidity and the proportional rank in wealth score distribution [40] as follows:

$$CI\;=\;\frac{2}{n^{2}\;\overset{-}{y}}\;\sum_{i\;=\;1}^{n}y_{i}r_{i}$$ (1)

Where CI is the concentration index, $\overset{-}{y}$ is the mean of childhood morbidity, r_i_ is the cumulative proportion that each individual represents over the total population once the latter has been ranked by the distribution of wealth score. The values of CI are bounded between $\overset{-}{y}-1$ and $1-\overset{-}{y};\;\overset{-}{y}-1\;\leq CI\;\leq 1-\overset{-}{y}$ when y is dichotomous [42]. CI acquires a negative value when the curve lies above the line of equality, which indicates a disproportionately higher prevalence of childhood morbidity among the poor (i.e., pro-poor). A positive value of CI signifies a higher concentration of health indicators among the rich (i.e., pro-rich). There is no socio-economic inequality in the distribution of childhood morbidity (y) when the value of CI is zero, and the CC coincides with the 45° line. The dichotomous character of the childhood morbidity may result in unstable bounds in response to varying means; therefore, the normalized standard index was estimated to check the robustness of the estimation [43,44]. The statistical tests of dominance between CCs were employed to estimate dissimilarity in prevalence across time. In addition, using the methods outlined in Wagstaff et al. [42] and Oaxaca [45], regression analysis was used to decompose the socio-economic inequality in relation to childhood morbidity to identify the contribution of different independent variables between 1993/94 and 2014. When there is a linear association between health outcome (y) and a set of k explanatory variables x [41], this can be specified as follows:

$$y\;=\;\alpha +\sum_{k}\beta_{k}{\overset{-}{x}}_{k}+\varepsilon$$ (2)

Where the CI may be expressed as a weighted sum of the partial concentration indices for the explanatory variables of inequality, being the weight of the elasticity of y with respect to x_k_ [42]. The concentration index for y, simplify the CI for y as

$$y\;=\;\sum_{k}\left(\frac{\beta_{k}{\overset{-}{x}}_{k}}{\overset{-}{y}}\right){CI}_{k}+\frac{G{CI}_{\varepsilon }}{\overset{-}{y}}$$ (3)

where $\overset{-}{y}$ is the mean of y, ${\overset{-}{x}}_{k}$ is mean x_k_, *CI*_*k*_ is a CI for x_k_ and *GCI*_*ε*_ is the generalized CI for the error term (*ε*) reflecting the inequality that is not explained by the systematic variation in the determinates across childhood morbidity. Eq (3) demonstrate that CI is equal to the weighted sum of CIs of the k determinants, where the weight for x_k_ is the elasticity of y with respect to x_k_. The relative contribution of a cause to the CI of childhood morbidity was fundamentally the product of its entity CI of childhood morbidity with respect to that particular cause [42]. Moreover, changes of CI over time (ΔCI = CI_t_—CI_t-1_) may be disentangled by using an Oaxaca-type decomposition [45] such that variation of CI can be explained by changes in elasticities and CI_k_,

$$\Delta CI\;=\;\sum_{k}\left(\frac{\beta_{k,t-1}{\overset{-}{x}}_{k,t-1}}{{\overset{-}{y}}_{t-1}}\right)({CI}_{k,t}-{CI}_{k,t-1})+\sum_{k}{CI}_{k,t}(\frac{\beta_{k,t}{\overset{-}{x}}_{k,t}}{{\overset{-}{y}}_{t}}-\frac{\beta_{k,t-1}{\overset{-}{x}}_{k,t-1}}{{\overset{-}{y}}_{t-1}})+\;\Delta \frac{{GCI}_{et}}{{\overset{-}{y}}_{t}}$$ (4)

Conversely, since Oaxaca decomposition is not unique [42], the variation of CI may be represented as:

$$\Delta CI\;=\;\sum_{k}\left(\frac{\beta_{k,t-1}{\overset{-}{x}}_{k,t-1}}{{\overset{-}{y}}_{t-1}}\right)({CI}_{k,t}-{CI}_{k,t-1})+\;\sum_{k}{CI}_{k,t}(\frac{\beta_{k,t}{\overset{-}{x}}_{k,t}}{{\overset{-}{y}}_{t}}-\frac{\beta_{k,t-1}{\overset{-}{x}}_{k,t-1}}{{\overset{-}{y}}_{t-1}})+\Delta \frac{{GCI}_{et}}{{\overset{-}{y}}_{t}}$$ (5)

When the childhood morbidity is dichotomous, probit models are optimal to perform all estimations. Then, some linear approximations are required to perform the decomposition analysis. This can be done by substituting in Eqs (2)–(4) β_k_ coefficients by *β*^*m*^_*k*_, which are the partial effects (dy/dx_k_) evaluated at sample means. In addition, when the outcome variable is dichotomous, the concentration index has to be corrected in order to allow comparisons between group of individuals from different time periods that may show different levels of use of health services [46]. In the context of a dichotomous outcome variable, the Erreygers’s CI is the CI multiplied by four times the mean health or outcome of interest [46]. Erreygers’ suggested corrected CI can be expressed as:

$$E\;=\;\frac{4\times \overset{-}{y}}{y^{max}-y^{min}}\;CI$$ (6)

Where *y*^*max*^ and *y*^*mix*^ are the bounds of y (childhood morbidity). When the Erreygers’ corrected index is used, the decomposition of inequality may be expressed as:

$$E\;=\;4\;\times \;\sum_{k}(\beta_{k}^{m}{\overset{-}{x}}_{k}){CI}_{k}+G{CI}_{\varepsilon }$$ (7)

This estimate produces an index that satisfies various attractive axiomatic properties for an inequality index, including the sign condition, scale invariance and mirror properties [47,48]. The adjusted CI method allows for an examination of the causes of (and their corresponding contributions to), and levels of changes in, childhood morbidity inequalities [41]. All statistical analyses were performed using Stata/SE-13 software (StataCorp, College Station, TX, USA). All the estimates were considered by sampling weights.

## Results

### Trends of socio-economic inequalities in childhood morbidity

**Fig 2:**
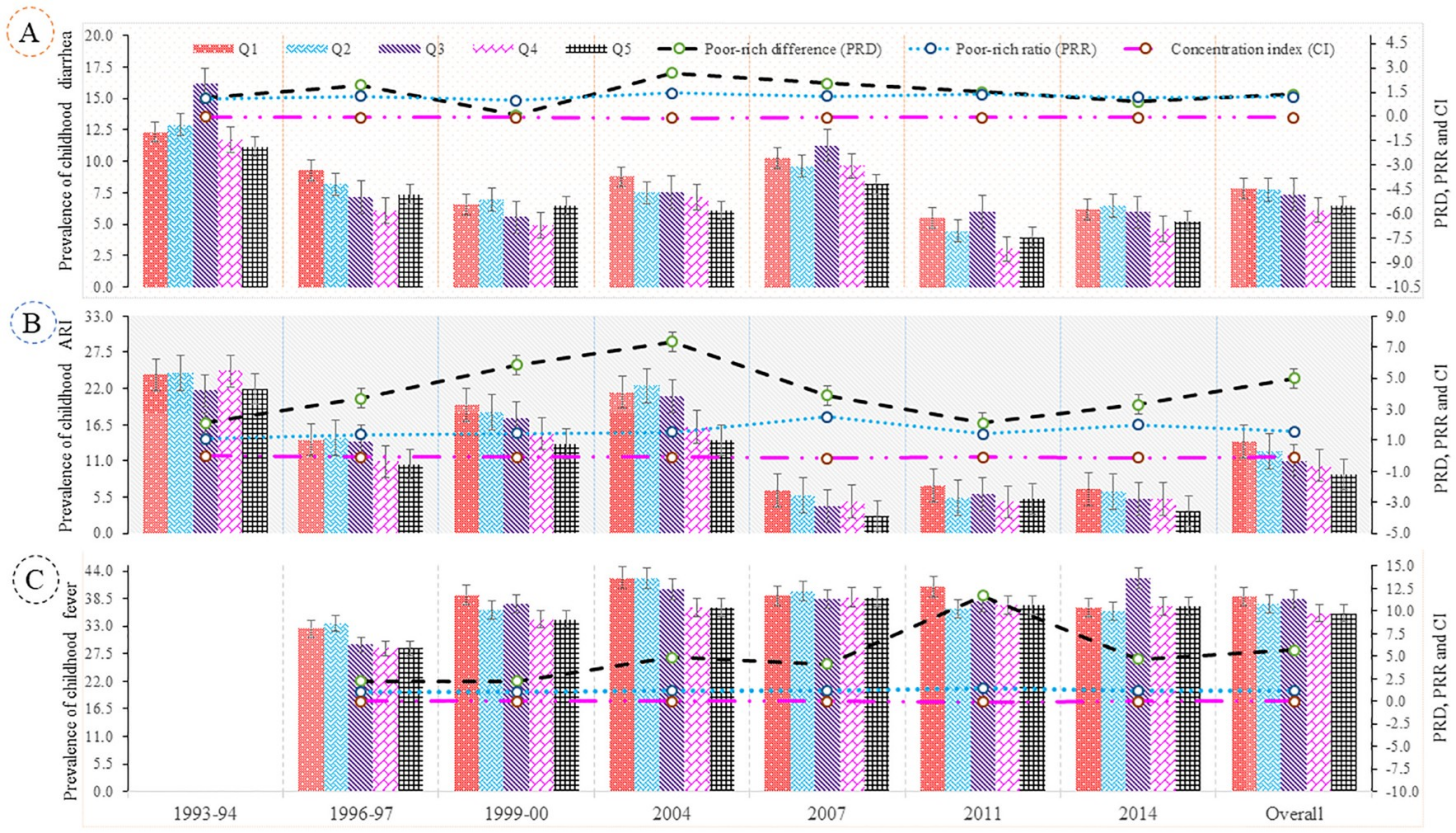
Distribution of childhood morbidity across scio-economic groups, 1993–2014. (A) Expressing the prevalence of childhood diarrhea and inequalities in terms of poor-rich difference (PRD), poor-rich ratio (PRR) and Erreygers’s concentration index (CI) (B) Presenting the prevalence of childhood acute respiratory infection (ARI) and inequalities in terms of PRD, PRR and CI (C) Showing the prevalence of childhood fever and inequalities in terms of PRD, PRR and CI.

Fig 2 depicts the trend in under-five childhood morbidity stratified across wealth quintiles. The overall prevalence of childhood morbidity was consequently highest for those living in socio-economically disadvantaged households (first quintile, Q_1_). The prevalence of diarrhea, ARI and fever were declined across the wealth quintiles from 1993/94 (1996/97) to 2014, and the rate of reduction was consequently highest for those living in the richer and richest families (upper wealth quintiles). In the poorest quintile, the prevalence of childhood diarrhea halved and ARI declined by 75% from 1993/94 to 2014. The absolute difference in prevalence of childhood diarrhea declined dramatically in the top quintile. On the other hand, the prevalence of childhood fever trended upward for all quintiles between 1996/97 and 2014.

**Fig 3:**
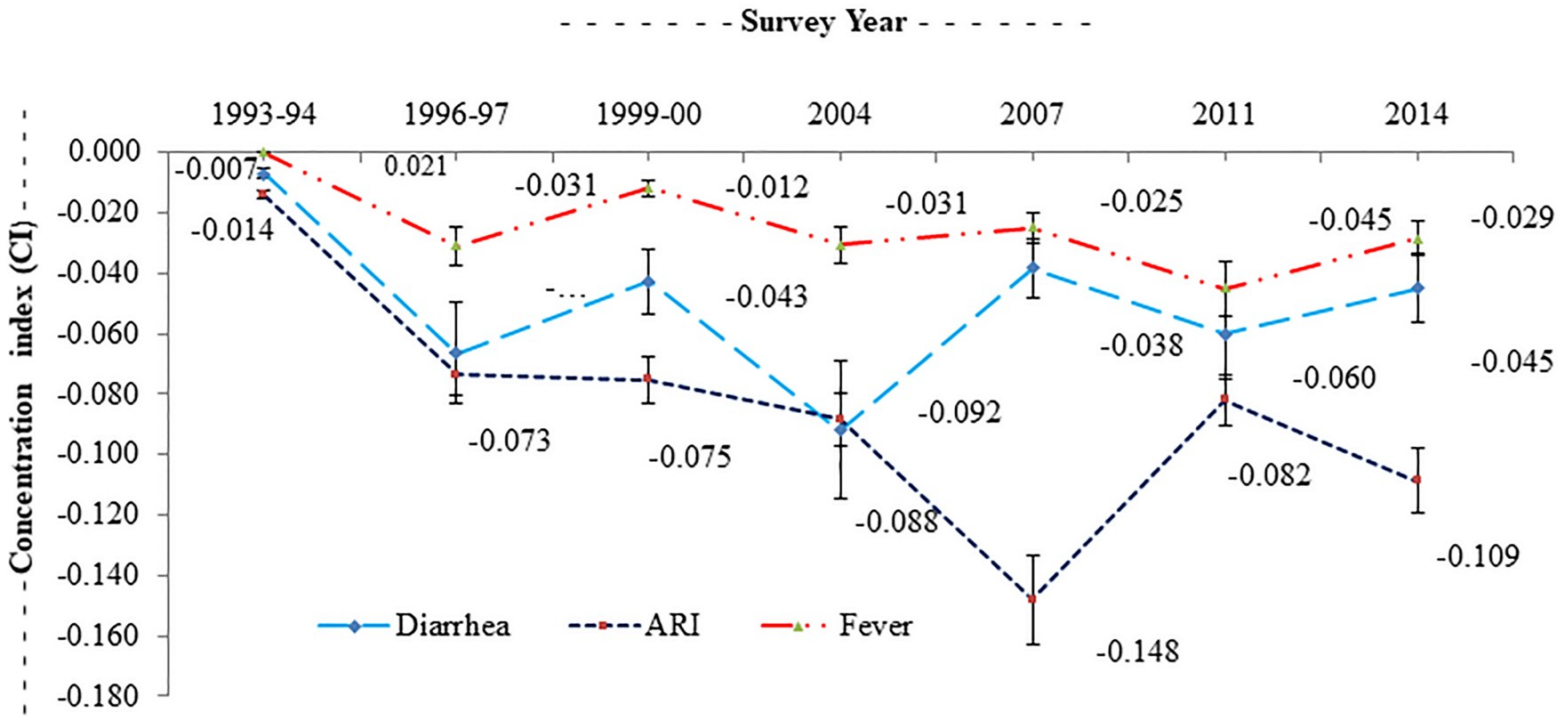
Trend of inequity in childhood morbidities, 1993–2014. The concentration curves (CC) for diarrhea, ARI, and fever in 1993/94 (1996/97 for fever) and 2014 (Fig 4).

While the overall prevalence of childhood diarrhea, ARI, and fever has fallen in recent times, poorer children are bearing an increasing share of the burden compared with higher wealth quintiles. For example, a poor-rich ratio (PRR) of 1.21 for children implies that the childhood diarrhea rate in the poorest quintile was about 1.21 times higher than that of the richest quintile. Similarly, the PRR due to ARI was 1.56, whereas this ratio was 1.17 for fever. The overall values of the CI for childhood morbidity for diarrhea, ARI and fever were -0.053, -0.088 and -0.029 respectively (Fig 3). This result showed that childhood morbidities (e.g., diarrhea, ARI, and fever) have a significantly higher concentration among the most disadvantaged group of children compared to the advantaged counterpart. Consistent with the results above, the CI for the prevalence of childhood diarrhea, ARI, and fever increased in absolute value from 1993/94 to 2014, which signified that children living in poverty bore a disproportionately higher share of the burden of these infectious diseases. The negative values indicated a significantly higher magnitude of socioeconomic inequality in childhood morbidity over the period.

**Fig 4:**
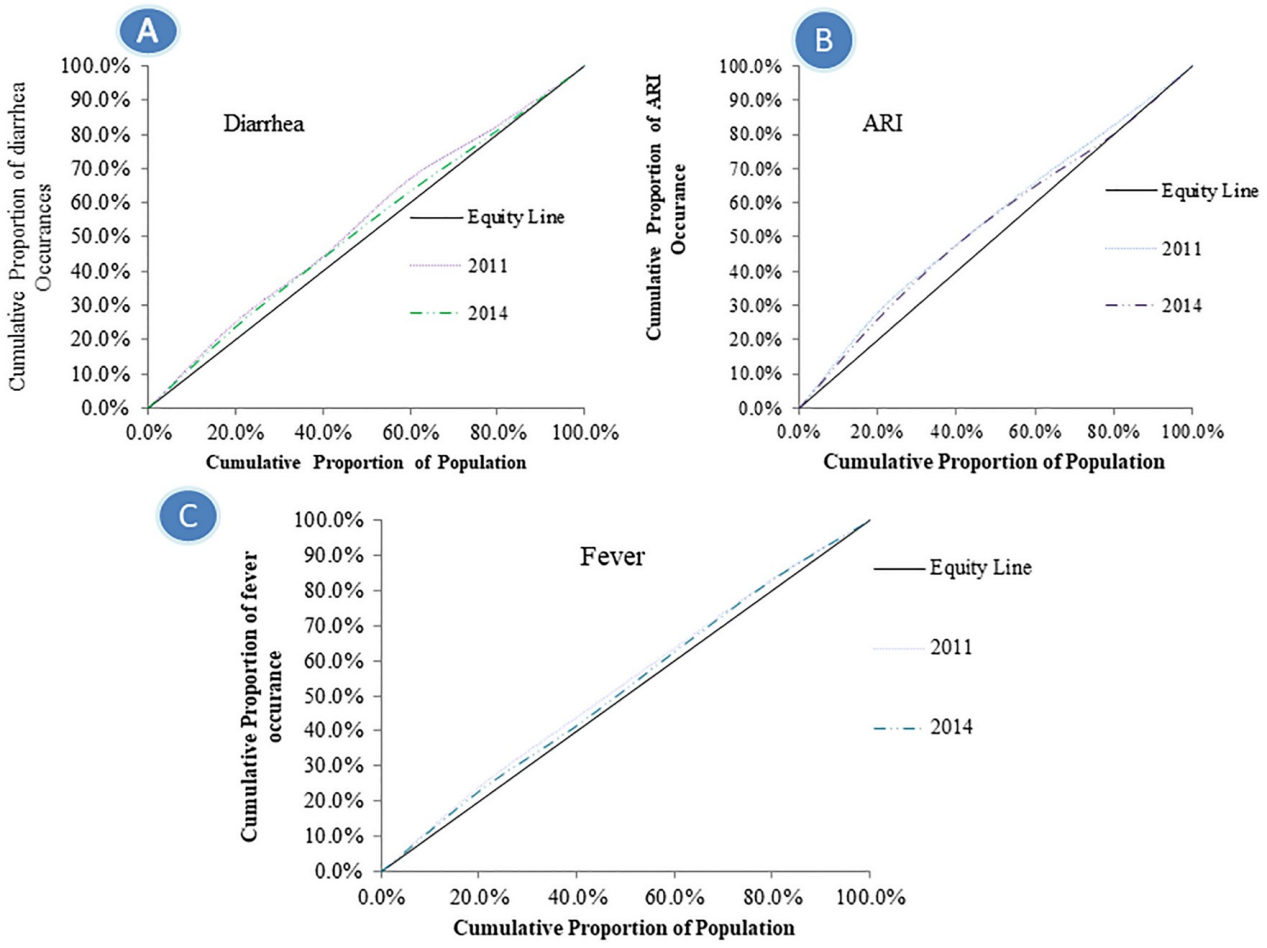
Concentration curve for childhood morbidities over 2011 to 2014. (A) Explicating childhood diarrhea (B) Uttering childhood acute respiratory infection (ARI) (C) Demonstrating childhood fever.

During both times, for three childhood morbidities, the CC was consistently above the line of equality, indicating a disproportionate concentration of the prevalence of childhood morbidity among poorer households. This result also signified a greater magnitude of socio-economic inequality in relation to the prevalence of diarrhea and ARI than the CC for fever (Fig 4). Expanding the degree of socio-economic inequalities between poor and rich quintiles made this even more apparent for childhood diarrhea, ARI, and fever, with the bottom third of the distribution experiencing 50% of the burden. In all cases, we may reject the null of non-dominance (P<0.01) using the test outlined in O’Donnell and Wagstaff [41,49]. The overall trend of inequality of childhood morbidity slowly declined over the period.

### Decomposition of childhood morbidity inequalities

Some variables were consistently associated with childhood diarrhea, ARI and fever in both 1993/94 (1996/97) and 2014 (Table 1). Mother’s education level was a statistically significant determinant for childhood diarrhea, ARI and fever. Children of higher educated mothers were less prone to diarrhea, ARI and fever. Furthermore, pre-delivery care, mothers’ breastfeeding practice, adverse chronic childhood undernutrition, access to health facility and mass media communication, and high coverage of immunisation were evident in explaining childhood diarrhea, ARI and fever. These factors were significantly associated with reducing childhood morbidity over the period 1993/94 or 1996/97-2014. In addition, household wealth status was also a predominant factor influencing childhood morbidities. Overall, children in affluent households have a lower probability of being exposed to morbidity. Moreover, there was a higher elasticity in terms of child’s age, mother’s schooling, pre-delivery care, parity, mother’s breastfeeding practice, chronic undernutrition status, access to health facility and mass media communication, high coverage of immunisation, and household wealth status for both years. The values of the elasticity suggested that these factors have become more significant in determining childhood adverse health outcomes in terms of diarrhea, ARI and fever.

**Table 1:** Inequality decomposition of the Erreygers’s concentration index for childhood morbidity, 1993–2014.

Variables	Coefficients & p-value				Elasticities		Erreygers concentration index (CI)		Contributions to Erreygers CI (%)		
	1993–94 (1996–97)^1^		2014		1993–94 (1996–97)^1^	2014	1993–94 (1996–97)^1^	2014	1993–94 (1996–97)^1^	2014	Change
**For diarrhea**											
Child’s age (months)	0.00	0.00	0.00	0.00	1.26	1.56	-0.26	-0.16	10.01	8.21	-1.80
Child’s age squared	0.07	0.02	0.05	0.02	-0.78	-1.86	-0.09	-0.08	7.21	-6.10	-13.31
Child sex (= male)	-0.04	0.33	0.11	0.09	-0.03	0.06	-0.02	-0.11	-4.65	-2.07	2.58
Mother age (years)	-0.03	0.00	-0.01	0.01	-0.73	-0.20	-0.14	0.12	-5.51	-10.42	-4.91
Mother schooling (years)	-0.02	0.67	-0.05	0.06	-0.02	0.08	0.19	0.10	12.10	11.15	-0.95
Birth order	0.08	0.00	0.04	0.05	0.26	0.90	-0.12	-0.17	10.37	14.03	3.66
Preceding birth interval (months)	0.02	0.42	-0.01	0.03	0.08	-0.03	0.22	0.08	-2.20	-7.29	-5.09
Antenatal care visits	-0.08	0.22	-0.03	0.05	-0.11	-0.05	0.13	0.15	15.01	12.31	-2.70
Households size	0.02	0.11	0.02	0.02	-0.12	0.11	0.09	0.09	2.71	3.17	0.46
Breastfeeding practice (= yes)	-0.03	0.01	-0.09	0.05	-0.05	0.13	0.06	0.08	6.92	6.22	-0.70
Access to health facility (= yes)	0.04	0.09	0.03	0.02	-0.07	0.06	-0.08	-0.05	3.56	5.26	1.70
Mass media communication (= yes)	-0.05	0.00	-0.09	0.00	0.08	0.09	0.05	0.03	2.06	2.19	0.13
Chronic undernutrition status (= yes)	-0.05	0.01	-0.16	0.01	0.14	0.13	-0.16	-0.09	13.96	13.10	-0.86
Vaccination coverage (= yes)	-0.13	0.02	-0.28	0.02	-0.03	0.01	-0.05	-0.04	-8.20	-9.13	-0.93
Residence (= rural)	0.06	0.05	0.14	0.09	0.01	0.02	0.06	0.04	-6.19	-5.01	1.18
Wealth index	-0.03	0.04	-0.04	0.04	0.07	-0.13	0.44	0.51	33.17	39.17	6.00
Total									90.33	74.79	-15.54
**For acute respiratory infection (ARI)**											
Child’s age (months)	0.01	0.00	- 0.03	0.04	-0.24	-0.96	-0.07	-0.29	22.11	17.21	-4.90
Child’s age squared	0.00	0.05	0.00	0.08	0.19	0.90	-0.08	-0.11	-8.09	-4.12	3.97
Child sex (= male)	0.21	0.04	0.19	0.08	0.11	0.10	-0.01	0.18	-6.89	-20.19	-13.30
Mother age (years)	0.00	0.01	- 0.00	0.01	0.01	-0.03	-0.27	-0.12	-6.86	-10.52	-3.66
Mother schooling (years)	-0.08	0.09	- 0.07	0.06	-0.05	-0.11	0.25	0.13	15.21	19.84	4.63
Birth order	-0.01	0.01	0.01	0.04	-0.03	0.03	-0.28	-0.14	8.79	12.31	3.52
Preceding birth interval (months)	0.01	0.02	- 0.02	0.03	0.03	-0.09	0.14	0.10	-2.29	-8.69	-6.40
Antenatal care visits	0.12	0.09	0.00	0.05	0.16	-0.01	0.29	0.19	19.01	18.31	-0.70
Households size	-0.02	0.01	- 0.04	0.02	-0.16	-0.23	0.20	0.15	14.66	16.50	1.84
Breastfeeding practice (= yes)	-0.02	0.04	-0.07	0.03	-0.04	0.11	0.07	0.12	6.27	10.11	3.84
Access to health facility (= yes)	0.05	0.06	0.06	0.01	-0.06	0.03	-0.06	-0.07	4.52	6.21	1.69
Mass media communication (= yes)	-0.03	0.01	-0.08	0.02	0.05	0.06	0.04	0.06	6.10	5.10	-1.00
Chronic undernutrition status (= yes)	-0.08	0.05	-0.13	0.03	0.04	0.04	-0.07	-0.11	5.09	5.11	0.02
Vaccination coverage (= yes)	0.01	0.08	- 0.17	0.16	0.00	-0.01	-0.06	-0.04	-5.12	-7.03	-1.91
Residence (= rural)	0.09	0.06	0.00	0.10	0.01	0.00	0.23	0.25	-8.10	-15.09	-6.99
Wealth index	-0.01	0.06	- 0.06	0.00	-0.04	-0.18	0.38	0.35	29.71	35.19	5.48
Total									94.12	80.25	-13.87
**For fever**											
Child’s age (months)	-0.01	0.00	0.01	0.00	-0.15	0.28	-0.17	-0.19	20.42	10.01	-10.41
Child’s age squared	0.00	0.00	0.00	0.01	-0.04	-0.44	-0.08	-0.04	-6.19	-3.15	3.04
Child sex (= male)	-0.01	0.04	0.01	0.03	-0.01	0.01	0.01	0.04	-6.67	-9.72	-3.05
Mother age (years)	-0.02	0.01	-0.01	0.08	-0.40	-0.15	-0.17	0.12	-4.61	-11.51	-6.90
Mother schooling (years)	0.04	0.03	-0.01	0.01	0.03	-0.01	0.12	0.09	12.98	11.35	-1.63
Birth order	0.04	0.02	0.04	0.03	0.12	0.10	-0.20	-0.35	9.73	7.31	-2.42
Preceding birth interval (months)	0.04	0.02	-0.03	0.02	0.13	-0.12	0.08	0.13	-5.15	-6.29	-1.14
Antenatal care visits	-0.02	0.05	0.03	0.03	-0.02	0.06	0.14	0.11	11.21	12.09	0.88
Households size	-0.02	0.01	-0.01	0.01	-0.11	-0.08	0.08	0.09	3.22	9.17	5.95
Breastfeeding practice (= yes)	-0.06	0.03	-0.06	0.03	-0.07	0.11	0.09	0.11	10.19	15.02	4.83
Access to health facility (= yes)	0.06	0.06	0.05	0.04	-0.09	0.09	-0.05	-0.06	4.15	4.16	0.01
Mass media communication (= yes)	-0.03	0.02	-0.07	0.01	0.10	0.08	0.06	0.02	3.60	5.11	1.51
Chronic undernutrition status (= yes)	-0.04	0.04	-0.10	0.04	0.06	0.11	-0.07	-0.08	9.09	14.12	5.03
Vaccination coverage (= yes)	-0.15	0.06	-0.39	0.00	-0.02	-0.02	-0.11	-0.04	-8.11	-7.13	0.98
Residence (= rural)	0.05	0.07	0.05	0.04	0.01	0.02	0.07	0.08	-4.19	-6.19	-2.00
Wealth index	-0.04	0.02	-0.02	0.04	-0.12	-0.07	0.23	0.31	40.78	32.17	-8.61
Total									89.45	83.52	-5.93

Note: 1996–97 for fever due to paucity of data. Coefficients and p-values were derived using probit regression model.

In comparing 1993/94 and 2014, variables that significantly contributed to childhood morbidity inequality across surveys include, mother schooling, pre-delivery care, household wealth, access to health facility and mass media communication, chronic undernutrition as well as immunisation coverage. Significantly, they accounted for more than 90% of the concentration index estimates for diarrhea, ARI and fever in 1993/94, which reduced to 75% by 2014. The total change in CI between 1993-94/ (1996–97 for fever) and 2014 was then decomposed (Table 2). Household wealth status was the most significant predictor explaining the increase in diarrhea, ARI and fever. Indeed, about 36% of the change in CI could be quantified by the wealth index for diarrhea, and about 40% for ARI and 39% for fever. Childhood chronic undernutrition was another important explanatory factor of being exposed to childhood diarrhea, contributing about 19% of the observed change in CI and 11% and 19% for ARI and fever, respectively. Similarly, pre-delivery care, breastfeeding practice, mother schooling, access to health facility and mass media communication and immunization coverage emerged as significant and substantial contributors to the rises in CI. However, the residual (14% for diarrhea, 20% for ARI, and 18% for fever) suggests that there are other unexplained predictors underlying the increase in CI of childhood morbidity.

**Table 2:** Oaxaca-type decomposition for change in inequality for childhood morbidity, 1993–2014.

Variables	E: Elasticity, CI:Concentration Index, t:2014, (t-1):1993–94 or (1996–97 for fever)					
	Variation (1)		Variation (2)		Total	
	E_t_ (CIt−CI_t-1_)	CI_t-1_ (E_t_−E_t-1_)	E_t-1_ (CIt−CI_t-1_)	CI_t_ (E_t_−E_t-1_)	Total	%
**For diarrhea**						
Child’s age (months)	-0.001	0.040	-0.005	-0.038	-0.001	12.00
Child’s age squared	0.002	-0.079	0.003	0.065	-0.002	-7.22
Child sex (= male)	0.002	0.090	0.010	-0.007	0.014	-9.20
Mother age (years)	0.009	0.051	-0.018	-0.009	0.038	-18.52
Mother schooling (years)	-0.043	-0.005	0.005	0.053	0.093	19.66
Birth order	-0.001	-0.047	-0.012	-0.016	-0.019	9.13
Preceding birth interval (months)	-0.002	-0.109	0.008	-0.016	-0.030	-5.29
Antenatal care visits	-0.020	0.030	-0.007	0.038	0.010	17.84
Households size	-0.002	-0.032	0.002	-0.081	-0.028	7.03
Breastfeeding practice (= yes)	-0.004	-0.002	-0.003	-0.002	-0.003	9.85
Access to health facility (= yes)	-0.003	-0.001	-0.005	-0.001	-0.003	3.56
Mass media communication (= yes)	-0.006	0.007	-0.004	-0.007	-0.003	4.62
Chronic undernutrition status (= yes)	-0.008	0.009	-0.007	0.012	0.012	19.11
Vaccination coverage (= yes)	-0.009	0.009	0.014	-0.013	-0.002	-6.15
Residence (= rural)	-0.067	-0.083	0.093	-0.002	-0.015	-5.70
Wealth score	-0.016	-0.034	0.076	-0.079	-0.013	35.48
Residual					-0.008	13.80
Total	-0.169	-0.156	0.150	-0.103	-0.078	
**For ARI**^a^						
Child’s age (months)	-0.002	-0.065	0.001	-0.046	-0.028	18.12
Child’s age squared	0.001	0.034	-0.001	0.023	0.014	-8.45
Child sex (= male)	0.004	0.006	0.002	0.063	0.019	-25.29
Mother age (years)	0.001	0.004	0.004	0.004	0.003	-19.59
Mother schooling (years)	0.013	-0.013	0.009	-0.014	-0.021	14.35
Birth order	0.005	0.003	0.001	0.003	0.033	23.68
Preceding birth interval (months)	-0.001	-0.033	0.003	-0.033	-0.016	-8.69
Antenatal care visits	0.008	0.003	-0.001	0.023	0.028	19.87
Households size	-0.001	-0.045	0.009	-0.046	-0.021	17.18
Breastfeeding practice (= yes)	-0.014	-0.002	-0.005	-0.006	-0.007	9.83
Access to health facility (= yes)	-0.021	0.011	-0.007	-0.005	-0.006	8.01
Mass media communication (= yes)	-0.008	0.007	-0.006	-0.006	-0.003	6.51
Chronic undernutrition status (= yes)	-0.003	0.003	-0.008	0.001	0.011	11.03
Vaccination coverage (= yes)	-0.009	-0.001	-0.001	-0.001	-0.003	-19.03
Residence (= rural)	-0.047	-0.011	-0.009	-0.017	-0.021	-6.09
Wealth score	-0.020	-0.040	-0.030	-0.096	-0.047	40.15
Residual					-0.006	20.41
Total	-0.094	-0.139	-0.039	-0.144	-0.089	
**For fever**						
Child’s age (months)	-0.001	-0.055	-0.001	-0.028	-0.021	15.13
Child’s age squared	-0.046	-0.084	0.006	0.051	-0.018	-7.19
Child sex (= male)	0.003	0.017	0.001	-0.008	0.003	-19.16
Mother age (years)	-0.003	-0.007	0.001	-0.008	-0.004	-12.51
Mother schooling (years)	0.023	0.091	0.017	0.054	0.026	9.77
Birth order	-0.002	0.011	0.013	-0.012	0.033	19.54
Preceding birth interval (months)	0.013	-0.042	0.002	-0.015	-0.011	-7.29
Antenatal care visits	0.025	0.075	-0.036	0.040	0.026	16.31
Households size	0.016	0.088	-0.016	-0.081	0.012	11.17
Breastfeeding practice (= yes)	-0.011	-0.009	-0.007	-0.003	-0.008	9.83
Access to health facility (= yes)	-0.020	0.008	-0.008	-0.015	-0.009	8.01
Mass media communication (= yes)	-0.008	0.006	-0.001	-0.021	-0.006	6.51
Chronic undernutrition status (= yes)	-0.007	0.002	-0.004	0.008	0.021	19.03
Vaccination coverage (= yes)	0.006	-0.031	-0.016	-0.012	-0.013	-8.33
Residence (= rural)	-0.019	-0.027	0.024	-0.002	-0.006	-8.19
Wealth score	-0.014	-0.096	-0.081	-0.072	-0.066	39.17
Residual					-0.009	18.20
Total	-0.045	-0.053	-0.107	-0.124	-0.082	

Note: Variation (1) (2) uses E_t_ (E_t-1_) and C_t_ (C_t-1_) to weight changes in C and E respectively. See Wagstaff et al. (2003) [42],

^a^ARI = Acute Respiratory Infections

## Discussion

Bangladesh has made remarkable progress in population health improvements over the past couple of decades [2,3,50]. However, socio-economic inequality is still a major contributor to childhood morbidity in Bangladesh. This study has examined the trend of inequality in childhood morbidity, especially for diarrhea, ARI and fever over a 22-year period and decomposed changes to identify potential risk factors that may explain the observed socio-economic inequality. Childhood morbidity overall showed a steady decline; however, such a decline was disproportionately distributed with regards to childhood morbidity, with the rate of reduction in the richest wealth quintile significantly outpacing that of the poorest. Consequently, the high degree of socioeconomic inequality persisted, based on study estimates. According to CC and CI analyses, poor-rich improvements were even more pronounced in the distribution of childhood diarrhea, ARI and fever. The analysis has demonstrated a range of characteristics in the trends. Children of disadvantaged socio-economic households were found to bear a greater burden of morbidity than their counterparts from advantaged households, confirming findings reported by others [8,51–53]. The findings of the decomposition analyses suggested that socio-economic inequality was the most dominant predictor driving inequalities in childhood morbidities. As part of the present national priorities, initiatives should therefore be considered that aim to reduce socio-economic inequalities in adverse childhood health outcomes (e.g., diarrhea, ARI and fever) as these must be addressed urgently. This will necessitate targeting underprivileged people with specific health interventions, aimed at ensuring accessibility and affordability of adequate health care services in relation to protection and promotion of maternal and child health. In addition, considering a societal perspective, policy efforts should be explored that mitigate structural factors such as unequal wealth distribution by focusing on effective financial mechanisms, social safety net programs, and creating employment opportunities along with impartial taxation, all of which might contribute to reducing inequalities in adverse child health outcomes. The decomposition analysis also revealed that the disparities in childhood morbidity were demonstrated by socio-economic inequalities in factors such as mother’s educational attainment, breastfeeding practice, chronic undernutrition status, access to health facility and mass media communication, reported pre-delivery care, and immunisation coverage.

This study found that childhood morbidity was more concentrated among the children of disadvantaged mothers with low levels of schooling. Some studies have shown that children whose mothers live in deprived households with low levels of schooling were disproportionately exposed to childhood morbidity [8,42,51–53]. These women have limited knowledge and practice in terms of childcare, nutrition, health communication, health services, hygienic drinking water, breastfeeding, and medical complications [38,42]. Pathogens are frequently transmitted through consuming food contaminated by unsafe and unaware preparation [38,54,55]. Furthermore, their physical and mental health conditions most likely place their children at a higher risk of morbidity [52,56–57]. Behavioural change through health promotion interventions could be an effective strategy to reduce the prevalence of childhood morbidity within poorer households and the children of mothers with low levels of schooling [42,51,55,58,59]. In addition, community awareness and mass media exposure might also inform mothers better about adverse child health outcomes.

Chronic malnourished children were significantly associated at a higher magnitude of the socioeconomic inequality regarding childhood diarrhea, ARIs and fever. This finding is consistent with relevant studies where malnourished or severely malnourished children significantly influenced the higher risk of childhood morbidity markers such as ARIs [60–62], persistent diarrhea [63–64] and fever [64,65]. One of the main reasons is that enteric pathogens are generally allied with childhood morbidity (e.g., diarrhea) among chronic malnourished children [64]. Frequent episodes of diarrhea and ARI in the absence of standard physical growth leads to malnutrition, which is associated with reduced neurodevelopmental outcomes [64,65]. For the economic viewpoint, household poverty may be a dominating factor of below living standard, accessing the quality of care, and chronic nutritional deficiency [62,66], wherein consequently leads to adverse outcomes such as chronic malnourishment of children, a weak immune system and, a resulting increased vulnerability to disease [60,67,68]. Although addressing general deficiency and socioeconomic inequality would lead to substantial reductions in undernutrition [69–73]. It should be a global priority, significant reductions in chronic malnutrition can also be made through the implementation of appropriate health and nutrition interventions to reduce their occurrence or ameliorate their adverse outcomes.

The results of this study showed that inadequate pre-delivery care among disadvantaged mothers was associated with high risk of childhood morbidity. This is consistent with previous findings that have shown that immature immune systems, due to lack of pre-delivery care in mothers and their children, result in more exposure to gastro-intestinal infections [6,53,58,66]. This finding signifies that mothers who had received a recommended number of pre-delivery care visits during pregnancy might be more protected against childhood morbidity compared to mothers who had received an inadequate number of visits. Non-vaccinated children within disadvantaged groups were also more exposed to childhood morbidity. Vaccination develops immunity systems in the human body, which have protective effects during childhood [54,59,67,68]. Thus, the findings suggested that the coverage of the Expanded Programme on Immunization (EPI) being formally launched in 1979 in Bangladesh needs to focus on the poorest, which will allow it to play a significant role in reducing adverse childhood outcomes (i.e., morbidity and mortality) [67,68].

### Strengths and limitations of the study

This present study has some important potential strengths. Data were derived through nationally representative seven round surveys that used standardised methods. This study considered three adverse under-five child health outcomes: diarrhea, ARI and fever. Regression-based decomposition methods were used to measure the magnitude of inequity in childhood morbidity over the 22-year period. The pooled method increased the study’s power and predicted socio-economic risk factors for childhood morbidity over time.

Some limitations of the study also exist. Although data were used to show changes in childhood morbidity over time, without longitudinal data of health coverage, it is not possible to observe the consequences for morbidity rates. Therefore, the study’s findings of observed changes in health and inequality should be interpreted with caution. All findings were generated based on individual self-reported data, which is an issue in terms of recall and social desirability biases. Recall bias and under-reporting of deaths may affect the estimated child morbidity rates. Future studies might confirm these results using better quality data. To overcome this limitation, data were pooled from multiple surveys over the 22-year period. Nevertheless, considerable sampling errors tend to happen where the number of observations is inadequate, necessitating caution when interpreting results. Finally, the nature of the cross-sectional study does not allow for exploring the causal inference of adverse child health outcomes.

## Conclusions and recommendations

The study results show that although childhood morbidity is decreasing over the study period, socioeconomic inequalities still persist. Policy makers should implement community-based health interventions targeting the significant influencing factors for the betterment of child health. This will also assist Bangladesh to achieve the targets of the Sustainable Development Goals. Reductions in under-five childhood morbidity have occurred nationally in Bangladesh. Some policy and strategic interventions in reproductive, maternal, newborn and child health issues may have contributed to this improvement. However, socio-economic inequality remains an ongoing issue for achieving universal health coverage in Bangladesh. This study results indicate that Bangladesh is lagging behind in terms of adolescent motherhood, poor maternal education, inadequate pre-delivery care, and low immunisation coverage. Yet, single interventions or strategies cannot necessarily be expected to make fundamental changes in the childhood morbidity in the short term. Public health interventions, targeting the socio-economically disadvantaged communities need to be implemented and such interventions should emphasise improving the health of women in reproductive age, healthy diet and nutrition, improved maternal and child health care, access to better health care services. This study may support policy makers in adopting new health policies to minimize inequalities in child health outcomes in Bangladesh.
