# Correction: Changes in inequality of childhood morbidity in Bangladesh 1993–2014: A decomposition analysis

**DOI:** 10.1371/journal.pone.0294378

**Published:** 2023-11-09

**Authors:** Rashidul Alam Mahumud, Khorshed Alam, Andre M. N. Renzaho, Abdur Razzaque Sarker, Marufa Sultana, Nurnabi Sheikh, Lal B. Rawal, Jeff Gow

There is an error in the affiliations for the first author. Specifically, Rashidul Alam Mahumud is not affiliated with #2.

Additionally, there was an error in the corresponding author’s listed email addresses. Correspondence regarding this article should only be sent to the following email address: rashed.mahumud@usq.edu.au.
